# Association between Ultraviolet B Exposure Levels and Depression in Taiwanese Adults: A Nested Case–Control Study

**DOI:** 10.3390/ijerph19116846

**Published:** 2022-06-03

**Authors:** Ci-Wen Luo, Shih-Pin Chen, Chen-Yu Chiang, Wen-Jun Wu, Chun-Jung Chen, Wen-Ying Chen, Yu-Hsiang Kuan

**Affiliations:** 1 Institute of Medicine, Chung Shan Medical University, Taichung 40201, Taiwan; kkjj88440@gmail.com (C.-W.L.); 007wu@csmu.edu.tw (W.-J.W.); 2 Department of Medical Research, Chung Shan Medical University Hospital, Taichung 40201, Taiwan; 3 Department of Internal Medicine, School of Medicine, Chung Shan Medical University, Taichung 40201, Taiwan; cshy752@csh.org.tw; 4 Department of Internal Medicine, Chung Shan Medical University Hospital, Taichung 40201, Taiwan; 5 Department of Veterinary Medicine, National Chung Hsing University, Taichung 40227, Taiwan; online70222@gmail.com (C.-Y.C.); wychen@dragon.nchu.edu.tw (W.-Y.C.); 6 Department of Education and Research, Taichung Veterans General Hospital, Taichung 40705, Taiwan; cjchen@vghtc.gov.tw; 7 Department of Pharmacology, School of Medicine, Chung Shan Medical University, Taichung 40201, Taiwan; 8 Department of Pharmacy, Chung Shan Medical University Hospital, Taichung 40201, Taiwan

**Keywords:** ultraviolet B (UVB), depression, nested case–control study

## Abstract

Depression is a common mental disorder that affects more than 264 million people worldwide. Anxiety, diabetes, Alzheimer’s disease, myocardial infarction, and cancer, among other disorders, are known to increase the risk of depression. Exposure to ultraviolet B (UVB) can cause human serotonin levels to increase. The vitamin D pathway is one mechanism through which ultraviolet light absorbed through the skin can affect mood; however, UVB exposure is known to increase the risk of cancer. In this study, we explored the effects of prolonged exposure to UVB on depression. Data were retrieved from the Taiwan National Health Insurance Research Database for 2008 to 2013. Each patient with depression was matched 1:4 with a comparison patient by sex and age (±5 years); thus, the study included 23,579 patients with depression and 94,316 healthy controls for comparison. The patients had been exposed to UVB for at least 1 year to observe the cumulative effect of UVB exposure. Based on the World Health Organization UV index, we divided the observation period data into five UV levels: low, moderate, high, very high, and extreme. A multivariate Poisson regression model was used to assess the risk of depression according to UVB exposure level, adjusting for sex, age, income, urbanization level, month, and comorbidities. The results revealed that the incidence rate ratio (IRR) for patients with depression was 0.889 for moderate levels (95% CI 0.835–0.947), 1.134 for high levels (95% CI: 1.022–1.260), 1.711 for very high levels (95% CI: 1.505–1.945), and 2.785 for extreme levels (95% CI: 2.439–3.180) when compared to low levels. Moderate levels of UVB lowered the risk of depression, while high levels of UVB gradually increased the risk. We propose that UVB at normal concentrations can effectively improve depression. However, exposure to high concentrations of UVB damage DNA results in physical diseases such as skin cancer, which increase the risk of depression.

## 1. Introduction

Two types of ultraviolet (UV) ray that reach the Earth’s surface are considered a major cause of skin disease: UVA and UVB. UVB rays have a shorter wavelength and higher energy than UVA rays, and they can directly damage human DNA, with strong evidence that they damage biological macromolecules, including lipids, proteins, and nucleic acids [1]. UVB rays also damage the outermost layer of skin, resulting in the formation of an active oxide; this excess reactive oxygen species are associated with skin diseases, such as skin cancer, skin aging, and damage to epidermal cells [2]. 

Depression is a common mental disorder, affecting more than 264 million people worldwide [3]. It is characterized by persistent grief and a lack of interest in activities that the patient previously enjoyed. Depression can also affect sleep and appetite and can cause fatigue and loss of concentration. These effects are long term or recurring, affecting an individual’s quality of life substantially [3] Depression is a primary cause of disability worldwide, exacerbating the global burden of disease [4]. Studies have demonstrated that anxiety, diabetes, Alzheimer disease, myocardial infarction, and cancer, among other diseases, increase the risk of depression [5,6]. However, 45.7% of people with severe depression have one or more anxiety disorders in their lifetime [7]. Evidence suggests a genetic risk between anxiety and depression; “neuroticism” is a common personality characteristic of those with anxiety and depression, and in terms of nerve conduction, the prefrontal transmission pathway is a common affective regulator of anxiety and depression [8]. The prevalence of depression in Cushing syndrome is 52.17%, and the level of cortisol in patients is a possible factor affecting depression [9,10]. The prevalence of depression in patients with Parkinson disease was reported to be 4 to 70%, whereas the prevalence of depression in those with Alzheimer disease has been reported to range from 0 to 86% and from 0 to 71% [11,12,13]. A previous study has reported that depression is a result of neurodegenerative diseases [14]. In patients with Alzheimer disease, the function of the brainstem mono–amine afferent nerves degrades, affecting the serotonin and locus cerulean in the secretion of norepinephrine [15]. Moreover, in Parkinson disease, the levels of 5–hydroxyindol acetic acid in the cerebrospinal fluid decreases, leading to nonmotor symptoms, including depression [16,17].

UVB exposure is known to increase the risk of cancer. Studies have reported that 13.7% of patients with cancer have depression after recovery (8.9% for adults without cancer, *p* < 0.001) [18]. However, exposure to UVB can cause human serotonin levels to rise [19]. One possible mood regulation effect of UV light absorbed through the skin is through the vitamin D pathway. The main source of vitamin D in the human body is skin exposure to sunlight (UVB 280–315 nm), which results in the conversion of 7–dehydrocholesterol to provitamin D3 [20]. Mechanisms by which serotonin is synthesized, released, and functions in the brain are regulated by vitamin D and omega–3 fatty acids, which include eicosapentaenoic acid (EPA) and docosahexaenoic acid (DHA). Brain serotonin is synthesized from tryptophan by tryptophan hydroxylase 2, which is transcriptionally activated by vitamin D via vitamin D receptors and vitamin–D–binding proteins [21,22,23]. Numerous studies have revealed that serotonin can inhibit depression, indicating that mood and depression may be directly affected by vitamin D deficiency and the influence this has on brain cells [24,25]. Approximately one–fifth of patients with CHD suffer from severe depression following diagnostic cardiac catheterization [26]. The study has shown vasoconstriction in patients with CHD, and an imbalance between angiotensin–II and angiotensin–(1–7) would be the potential risk factor for depression in patients [8]. Some scholars have proposed the monoamine hypothesis, which suggests that because neurotransmitters are involved in the regulation of neurons, pressure–induced serotonin, norepinephrine, and dopamine deficiencies in the synaptic cleft cause depressive symptoms. The lifetime incidence of depression in patients with HIV is between 22 and 45% [27]. A previous study has suggested that reductions in plasma cobalamin as a result of HIV increase the risk of depression [28]. The activation of mitotic pathways is induced by reactive oxygen species (ROS) generated by UVB, which then results in tissue inflammation and tumor generation via the activation of tumor promoters [29,30]. The evidence has reported the direct link between depression and skin disorders, especially facial skin disorders [31]. In patients with high–risk primary melanoma, 10% of patients develop symptoms of anxiety or depression. Furthermore, anxiety or depression resolves rapidly in patients after melanoma removal treatment [32,33]. Therefore, assessing the effects of UVB–induced anxiety and depression, as well as prognostic treatment after cancer, have become more clinically important. Relevant studies have demonstrated an association between depression and UVB, but a direct relationship between them has yet to be explored. Therefore, in this study, we explored the effects of prolonged exposure to UVB on depression.

## 2. Methods

### 2.1. Data Source

Taiwan’s National Health Insurance Research Database (NHIRD) contains insurance data on approximately 98% of Taiwan’s population, obtained from the National Health Insurance (NHI) program of the National Health Research Institutes. Since its establishment in 1995, the NHI program has provided comprehensive medical care to approximately 99% of Taiwan’s 23 million citizens and retains both outpatient and inpatient treatment records. The databases are de–identified regarding patient information and cannot be linked to other databases [34]. This study was approved by the Human Research Ethics Committee of the Institutional Review Board of Chung Shan Medical University Hospital (CS2–20075).

The data used in this study were derived from an NHIRD data subset, the Longitudinal Health Insurance Database 2000 (LHID2000), which contains data on all claims for 1 million representative beneficiaries randomly selected from the NHIRD from 2000 to 2013. The LHID2000 records the International Classification of Diseases, Ninth Revision, Clinical Modification (ICD–9–CM) codes for patient diagnosis and procedures, prescription details, and expenditure related to all outpatient and inpatient medical insurance claims.

The Ambient Air Quality Monitoring Network (AQMN) [35], administered by the Environmental Protection Agency of Taiwan, uses a cone–element oscillating microbalance (R&P 1400, Rupprecht and Patashnick, New York, NY, USA) to measure UVB exposure in the atmosphere and calculate the UV index (UVI); the hourly measured value is recorded through this method. In this study, months served as the unit of time measurement used here, with monthly average cumulative exposure being used as a measure of patient exposure [20]. The benchmark was based on the exposure recorded at the station in the patient’s residential area; if no station was located in the patient’s residential area, the nearest station was used as the benchmark. We excluded daily observations containing missing values for more than 8 h and monthly observations containing missing values for more than 10 days. The calculation formula for the average monthly UVB exposure is as follows: average monthly UVB exposure = (total UVI for all exposure days/numbers of exposure–day) × numbers of exposure–month. We used the data provided by AQMN to estimate the monthly average UVB exposure of patients and subsequently divided them into groups to examine the exposure–response relationship. With reference to World Health Organization (WHO) UVI exposure standards, the observation period data were divided into 5 levels: low (<3 UVI × numbers of exposure–month), moderate (≥3 to <6 UVI × numbers of exposure–month), high (≥6 to <9 UVI × numbers of exposure–month), very high (≥9 to <11 UVI × numbers of exposure–month), and extreme (≥11 UVI × numbers of exposure–month).

Patients with depression were identified using the ICD–9–CM codes 296.2x and 296.3x, for those with 2 or more visit records or more than one psychiatric hospitalization record. Only patients with records from between 2008 and 2013 were included in this study. The index date was the date on which the patient was first diagnosed with depression. 

### 2.2. Study Population

**Figure 1:**
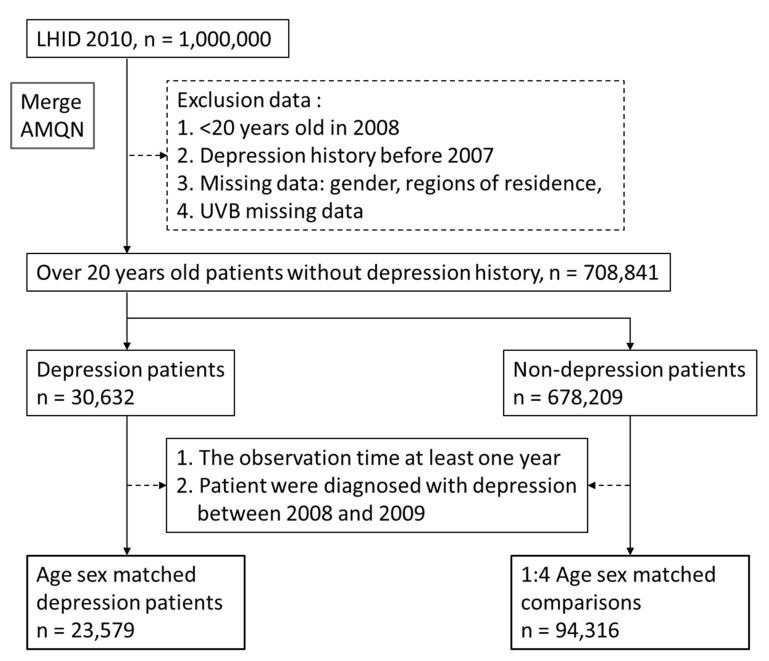
Study flowchart of Depression and comparisons.

This study was a nested case study. The individuals in the case group were patients diagnosed with depression (ICD–9–CM codes 296.2, 296.3, 300.4, and 311) [36] before 2013 and who did not have depression between 2008 and 2009; the comparison group consisted of individuals who had not been diagnosed with depression before 2013. Each patient in the case group was matched 1:4 with those in the comparison group by sex and age (±5 years). Thus, the cases (depression patients) and comparisons consisted of 23,579 and 94,316 people, respectively. All those included must have been exposed to UVB for at least 1 year since 2008 for the cumulative UVB exposure to be calculated (Figure 1). Patients with missing data and those who died before the age of 20 years were excluded. 

### 2.3. Comorbidities

Comorbid diabetes (ICD–9 CM code 250), anxiety (ICD–9 CM 300.00), Cushing’s disease (ICD–9 CM 255.0), Parkinson’s disease (ICD–9 CM 332, 332.0), Alzheimer’s disease (ICD–9 CM 331.0, 290.1), coronary heart disease (CHD, ICD–9 CM 410–414.99, 414.0, 414.9, 414.00), breast cancer (ICD–9 CM 174.9), and human immunodeficiency virus (HIV, ICD–9 CM 042) were regarded as confounding factors in the Poisson regression [37,38,39,40,41].

### 2.4. Statistical Analysis

We matched the patients with healthy controls in a 1:4 ratio according to sex, age, and index date, using known confounding factors to enhance their comparability. A Chi–squared test was used to compare categorical variables such as sex, income, and urbanization level between the case and comparison group, and a two–tailed *t*–test was used to identify differences between the two groups [42]. Variables that were used included age. The incidence density from the index date to 2013 and the monthly probability of depression were calculated. One–hundred person–months were used to observe the risk of depression per 100 people for 1 month or 1 person for 100 months. We used a multivariate Poisson regression model to assess the risk of each participant exposed to UVB. The duration of the period of depression was used as a detailed risk estimation to offset the relationship between UVB exposure level and depression risk. The results are expressed in terms of the incidence rate ratio (IRR) and 95% CI. All statistical analyses were performed using SAS v. 9.4 (SAS Institute, Cary, NC, USA) and *p* < 0.05 was considered statistically significant.

## 3. Results

No significant difference was observed between the participants in the comparison and case groups with regard to sex and age (Table 1). However, the proportion of women in the case group was higher, and the case group included more participants with a low income (64.25%) than did the comparison group (61.85%). Among those with depression, a significantly higher number of participants were low–income patients than those who were not. The majority of participants in both groups (case group: 33.15%; comparison group: 30.46%) lived in highly urbanized areas (*p* < 0.05). Regarding comorbidities, significant differences were observed in relation to diabetes, anxiety, Cushing disease, Parkinson disease, Alzheimer disease, coronary heart disease (CHD), breast cancer, and human immunodeficiency virus (HIV) status, with higher percentages in the case group (15.93, 41.03, 0.13, 2.26, 0.94, 14.08, 1.09, and 0.25%, respectively) than in the comparison group.

Table 2 summarizes the distributions of WHO UVB levels for the patients with depression and the comparison group, with 87,700 participants in the comparison group (mean UVI (SD) = 1.37 (0.26) UVI, median UVB = 1.29 UVI) and 21,486 in the case group (mean UVI (SD) = 1.43 (0.28) UVI, median UVB = 1.28 UVI) being exposed to low levels, with 3937 participants in the comparison group (mean UVB (SD) = 4.60 (0.73) UVI, median UVB = 4.67 UVI) and 1236 in the case group (mean UVB (SD) = 4.51 (0.80) UVI, median UVB = 4.60 UVI) being exposed to moderate levels, with 1223 participants in the comparison group (mean UVB (SD) = 6.86 (0.55) UVI, median UVB = 6.82 UVI) and 381 in the case group (mean UVB (SD) = 6.88 (0.57) UVI, median UVB = 6.87 UVI) being exposed to high levels, with 763 participants in the comparison group (mean UVB (SD) = 9.20 (0.81) UVI, median UVB = 9.16 UVI) and 381 in the case group (mean UVB (SD) = 9.32 (0.84) UVI, median UVB = 6.87 UVI) being exposed to very high levels, and with 693 participants in the comparison group (mean UVB (SD) = 13.87 (2.14) UVI, median UVB = 9.16 UVI) and 230 in the case group (mean UVB (SD) = 13.91 (2.31) UVI, median UVB = 12.09 UVI) being exposed to extreme levels. We used a two–tailed *t*–test to determine the differences in average monthly UVB exposure between the case and comparison groups for the continuous variables, which revealed significant differences. An χ^2^ test was used to assess the differences in WHO levels between the 2 groups, which also revealed significant differences.

Table 3 presents the risk of UVB levels and confounding variables for depression. We adjusted for gender, age, low income, urbanization, and comorbidities. There was no significant difference between gender and age after matching (results not shown). After performing the multiple Poisson regression of the WHO UVB levels and comparing the results with those for low levels, the IRR of moderate levels was 0.889 (95% CI: 0.835–0.947), for high levels it was 1.134 (95% CI: 1.022–1.260), for very high levels it was 1.711 (95% CI: 1.505–1.945), and for extreme levels it was 2.785 (95% CI: 2.439–3.180). In urbanization level, comparing the results with those for high levels, the IRR of moderate urbanization was 0.891 (95% CI: 0.863–0.920), emerging town was 0.733 (95% CI: 0.705–0.762), general town was 0.799 (95% CI: 0.764–0.835), aged township was 0.839 (95% CI: 0.756–0.931), agricultural town was 0.892 (95% CI: 0.824–0.966), and remote township was 0.748 (95% CI: 0.693–0.808). In comorbidities comparing the results without those disease, the IRR of diabetes was 1.138 (95% CI: 1.096–1.181), anxiety was 3.685 (95% CI: 3.587–3.785), Parkinson’s disease was 1.648 (95% CI: 1.509–1.799), Alzheimer’s disease was 1.685 (95% CI: 1.473–1.927), CHD was 1.205 (95% CI: 1.159–1.254), breast cancer was 1.280 (95% CI: 1.131–1.449), and HIV was 2.283 (95% CI: 1.763–2.955).

Table 4 lists the risk of UVB levels for depression in the subgroups according to the results of the multiple Poisson regression, which were then compared with the results for low levels. Each subgroup was adjusted for gender, age, low income, urbanization, and comorbidities. The results only show the risk of depression for each subgroup exposed to UVB. In female patients, the IRR of moderate exposure levels was 0.885 (95% CI: 0.816–0.959), for very high levels it was 1.697 (95% CI: 1.439–2.000), and for extreme levels it was 2.825 (95% CI: 2.381–3.352). In male patients, the IRR of moderate level was 0.899 (95% CI: 0.814–0.993), for high levels it was 1.305 (95% CI: 1.113–1.531), for very high levels it was 1.731 (95% CI: 1.411–2.123), and for extreme levels it was 2.749 (95% CI: 2.227–3.393). In low income patients, the IRR of moderate levels was 0.888 (95% CI: 0.817–0.966), for very high levels it was 1.823 (95% CI: 1.529–2.174), and for extreme levels it was 2.780 (95% CI: 2.300–3.359). In non–low income patients, the IRR of moderate levels was 0.884 95% CI: (0.805–0.972), for very high levels it was 1.593 (95% CI: 1.321–1.921), and for extreme levels it was 2.808 (95% CI: 2.331–3.383). For patients who didn’t live in highly urbanized areas, the IRR of moderate levels was 0.895 (95% CI: 0.844–0.949), for high levels it was 1.151 (95% CI: 1.040–1.275), for very high levels it was 1.742 (95% CI: 1.536–1.976), and for extreme levels it was 2.828 (95% CI: 2.482–3.223). In diabetes patients, the IRR of very high levels was 1.639 (95% CI: 1.204–2.231), and for extreme levels it was 2.683 (95% CI: 1.971–3.652). In non–diabetes patients, the IRR of moderate levels was 0.887 (95% CI: 0.827–0.951), for high levels it was 1.122 (95% CI: 0.999–1.260), for very high levels it was 1.730 (1.503–1.993), and for extreme levels it was 2.828 (2.441–3.276). In anxiety patients, the IRR of moderate levels was 0.804 (0.730–0.885), for very high levels it was 1.699 (1.407–2.052), and for extreme levels it was 2.821 (2.323–3.426). In non–anxiety patients, the IRR of moderate levels was 0.956 (95%CI: 0.881–1.038), for high levels it was 1.734 (95%CI: 1.457–2.065), and for very high levels it was 2.806 (95%CI: 2.339–3.365). In Parkinson’s disease patients, the IRR of extreme levels was 2.157 (95%CI: 1.179–3.945). In non–Parkinson’s disease patients, the IRR of moderate levels was 0.896 (0.84–0.954), for high levels it was 1.139 (1.024–1.268), for very high levels it was 1.727 (1.518–1.965), and for extreme levels it was 2.844 (2.482–3.259). In non–Alzheimer’s disease patients, the IRR of moderate levels was 0.896 (95%CI: 0.840–0.954), for high levels it was 1.139 (95%CI: 1.024–1.268), for very high levels it was 1.727 (95%CI: 1.518–1.965), and for extreme levels it was 2.844 (95%CI: 2.482–3.259). In coronary heart disease patients, the IRR of moderate levels was 0.824 (95%CI: 0.707–0.960), for very high levels it was 1.727 (95%CI: 1.294–2.304), and for extreme levels it was 2.108 (95%CI: 1.479–3.004). In non–coronary heart disease patients, the IRR of moderate levels was 0.901 (95%CI: 0.841–0.965), for high levels it was 1.137 (95%CI: 1.015–1.275), for very high levels it was 1.701 (95%CI: 1.474–1.962), and for extreme levels it was 2.929 (95%CI: 2.538–3.381). In breast cancer patients, the IRR of extreme levels was 4.894 (95%CI: 1.414–16.936). In non–breast cancer patients, the IRR of moderate levels was 0.889 (95%CI: 0.835–0.947), for high levels it was 1.140 (95%CI: 1.026–1.266), for very high levels it was 1.717 (95%CI: 1.509–1.952), and for extreme levels it was 2.770 (95%CI: 2.424–3.165), In HIV patients, the IRR of very high levels was 9.134 (95%CI: 1.196–69.763). In non–HIV patients, the IRR of moderate levels was 0.889 (95%CI: 0.835–0.947), for high levels it was 1.134 (95%CI: 1.021–1.259), for very high levels it was 1.709 (95%CI: 1.503–1.943), and for extreme levels it was 2.777 (95%CI: 2.431–3.172).

## 4. Discussion

In this study, an exposure to moderate levels of UVB lowered the risk of depression, whereas high levels gradually increased the risk. We therefore propose that UVB at normal concentrations can effectively mitigate depression, but exposure to high concentrations of UVB damages DNA, causing some physical diseases, including skin cancer, which then increase the risk of depression. 

Studies have demonstrated that women are at a higher risk of depression than men are, which is consistent with our results, which revealed more female patients with depression (Table 1) [43,44]. The monoamine hypothesis of depression is indicated to indicate that depression is caused by the deficiency of pressure–induced monoamine neurotransmitters, including serotonin, norepinephrine, and dopamine in the synaptic cleft [39]. Therefore, changes in monoamines can cause breast cancer to promote depression in patients, especially young women, who are more susceptible to the effects of dopamine and serotonin [39]. We discovered that the higher the urbanization level was, the higher the risk of depression was, which may be because people living in the city have more pressure from school and work, leading to higher levels of stress [45]. In addition, studies have demonstrated that a low income has a negative effect on health, which is consistent with our research results that associate low income with a higher risk of depression [46]. Life changes caused by illness require patients or family members to adapt to new conditions, which can easily lead to anxiety and depression. Therefore, stress–related illnesses often occur concurrently. According to the literature, approximately 15 to 20% of patients with diabetes have depression, which is closely related to the increase in mortality of those with diabetes. Moreover, patients with diabetes and depression often do not perform well with regard to the use of medication and diet control, leading to a vicious circle between diabetes and depression [47]. Furthermore, the results of these studies are consistent with ours, suggesting that our results are closer to reality after adjustment.

Regarding the effect of UVB on depression, we referred to other studies to perform a stratified analysis according to sex, income, degree of urbanization, and comorbidities, including diabetes, anxiety, and CHD [47,48]. Our study demonstrated that exposure to moderate UVB levels reduces the risk of depression, but high levels and above are an independent risk factor for depression. At present, we have purposed the direct relationship between UVB and depression. A previous study has revealed that the human body absorbs UVB through the skin, helping the body to synthesize vitamin D. Vitamin D can be synthesized in the skin through a photosynthetic reaction triggered by exposure to UVB radiation. Production efficiency depends on the number of UVB photons that penetrate the skin [49]. It starts with the production of 25–hydroxyvitamin D, the main form of vitamin D that circulates in the blood [50,51]. A cross–sectional study suggested that the association between 25–hydroxyvitamin D and depression was an inverted U–shaped curve with an inflection point of 56.2 nmol/L [52]. However, in a meta–analysis of another randomized controlled trial, vitamin D supplementation did not significantly reduce depression [53]. In a human study, the production of IL–1β and IL–6 is induced by UV exposure [54,55]. In a murine study, IL–6 may play the key role in altered skin immune responses via generation of IL–10 after UV exposure [56]. The proinflammatory cytokines including IL–6, tumor necrosis factor–alpha, and IL–1, are associated with the pathophysiology of major depressive disorder in the brain [57,58,59]. Furthermore, patients with major depressive disorder have elevated concentrations of IL–6 in their plasma when compared with people without depression [60,61,62]. Inadequate levels of vitamin D, EPA, or DHA caused serotonin activation and dysfunction during critical developmental periods and may be a potential mechanism for neuropsychiatric disorders and depression [11,14]. Vitamin D activates the tph2 enzyme, which is the second hydrogen source of human tryptophan. Oxidase is a key enzyme responsible for the synthesis of serotonin in the central nervous system. It can convert amino acids into serotonin, which, in turn, affects mood [14,15]. In several experiments on serotonin and depression, we discovered that the experimental time of 1 week to 1 month provided an insufficient exposure over time for testing the association. However, our results have shown that under lower concentrations of UVB exposure (for at least 1 year, including UVB low level and UVB moderate level), the risk of UVB for depression is reduced. These results are similar as previous studies [63,64]. In keratinocytes, UVB light stimulates ROS production. The lysates of these cells were found to have nondialyzable trypsin, which is able to influence UVB light to generate ROS. The accumulation of excess ROS through the action of catalase can lead to oxidative stress and DNA damage [65,66]. Therefore, we suggest that moderate exposure to lower levels of UVB or protection of UVB reduces the risk of depression. Therefore, direct exposure to high levels of UVB results in increased risk of depression.

This study has several limitations. We lacked information on the living and exercise habits of the patients, such as sleep quality and exercise frequency, but we were able to adjust for several comorbidities [67,68]. We also lacked information on the time patients spent indoors and outdoors, which could have influenced differences in UVB exposure. In addition, our UVB exposure data were based on the patients’ household registration area, but we were unable to confirm the actual residential area of the patient. Moreover, the patients’ degree of depression was unknown; we were only able to evaluate the effect on depression of different concentrations of UVB but not whether these concentrations would aggravate the degree of depression. 

## 5. Conclusions

In conclusion, the long–term exposure to moderate levels of UVB can effectively inhibit the development of depression, but as the UVB level increases, the risk of depression increases. Further experiments are required to identify the related mechanisms. In clinical practice, perhaps because of the risk–related diseases affected by UVB, more attention should be paid to the patient’s psychological state in medical treatment, to enable the patient to cope with the disease more actively and reduce the related risks caused by depression. Then, patients will be able to cope more actively with their disease and reduce the associated risk of depression due to UVB.

## Figures and Tables

**Table 1:** Baseline characteristics of participants of depression and comparison.

	Comparison		Depression		*p-*Value
	(*n* = 94,316)		(*n* = 23,579)		
**Gender**					
Female	57,884	(61.37%)	14,471	(61.37%)	1.0000
Male	36,432	(38.63%)	9108	(38.63%)	
**Age**					
Mean ± SD	47.81 ± 16.78		47.96 ± 17.03		0.2180
**Low income**					
Yes	58,336	(61.85%)	15,150	(64.25%)	<0.0001
No	35,980	(38.15%)	8429	(35.75%)	
**Urbanization level**					
Highly urbanized	28,730	(30.46%)	7816	(33.15%)	<0.0001
Moderate urbanization	28,273	(29.98%)	7330	(31.09%)	
Emerging town	18,369	(19.48%)	3684	(15.62%)	
General town	11,410	(12.1%)	2815	(11.94%)	
Aged Township	1563	(1.66%)	398	(1.69%)	
Agricultural town	2765	(2.93%)	806	(3.42%)	
Remote township	3206	(3.4%)	730	(3.1%)	
**Comorbidities**					
Diabetes	11,466	(12.16%)	3755	(15.93%)	<0.0001
Anxiety	8665	(9.19%)	9674	(41.03%)	<0.0001
Cushing’s disease	50	(0.05%)	30	(0.13%)	<0.0001
Parkinson’s disease	723	(0.77%)	533	(2.26%)	<0.0001
Alzheimer’s disease	263	(0.28%)	222	(0.94%)	<0.0001
Coronary heart disease	8568	(9.08%)	3319	(14.08%)	<0.0001
Breast cancer	689	(0.73%)	256	(1.09%)	<0.0001
Human immunodeficiency virus	41	(0.04%)	58	(0.25%)	<0.0001

Basic demographic characteristics.

**Table 2:** Baseline distribution of UVB of depression and comparison.

	Comparison						Depression						*Two-Tailed Test* *p*-Value	*Chi-Square* *p*-Value
	N (%)		Average Monthly UVB Exposure (UVI)				N (%)		Average Monthly UVB Exposure (UVI)					
			Mean (SD)	Q1	Median	Q3			Mean (SD)	Q1	Median	Q3		
**Total participants Ultra–violet Index WHO standard level**														
Low level	87,700	(92.99%)	1.37 (0.26)	1.21	1.29	1.53	21,486	(32.65%)	1.43 (0.28)	1.22	1.38	1.56	<0.0001	<0.0001
Moderate level	3937	(4.17%)	4.60 (0.73)	3.99	4.67	5.16	1236	(5.91%)	4.51 (0.80)	3.88	4.60	5.11		
High level	1223	(1.3%)	6.86 (0.55)	6.45	6.82	7.23	381	(2.78%)	6.88 (0.57)	6.45	6.87	7.31		
Very high level	763	(0.81%)	9.20 (0.81)	8.51	9.16	9.69	246	(2.43%)	9.32 (0.84)	8.54	9.40	10.00		
Extreme level	693	(0.73%)	13.87 (2.14)	12.02	13.63	15.15	230	(3.39%)	13.91 (2.31)	12.09	13.40	15.15		

UVB, ultraviolet radiation b; UVI, ultra–violet index; SD, Standard Deviation; WHO, world health organization, Q1, first quartile; Q3, third quartile.

**Table 3:** Poisson regression analysis of Ultraviolet radiation b (UVB) and depression.

	Observed	Incidence Density (95% CI)	Adjusted IRR (95%CI)
	Person-Months	Per 100 Person-Months	
**UVB WHO level (reference: Low level)**			
Low level	866,325	2.48 (2.47–2.49)	Reference
Moderate level	66,201	1.87 (1.84–1.89)	0.889 (0.835–0.947)
High level	15,396	2.47 (2.44–2.51)	**1.134 (1.022–1.260)**
Very high level	6845	3.59 (3.53–3.66)	**1.711 (1.505–1.945)**
Extreme level	4112	5.59 (5.47–5.73)	**2.785 (2.439–3.180)**
**Low-income (reference: No)**			
Yes	613,820	2.47 (2.45–2.48)	1.080 (1.052–1.110)
No	345,059	2.44 (2.42–2.46)	Reference
**Urbanization level (reference: Highly urbanized)**			
Highly urbanized	309,804	2.52 (2.50–2.55)	Reference
Moderate urbanization	297,639	2.46 (2.44–2.48)	0.891 (0.863–0.920)
Emerging town	151,564	2.43 (2.40–2.46)	0.733 (0.705–0.762)
General town	118,742	2.37 (2.34–2.40)	0.799 (0.764–0.835)
Aged Township	16,233	2.45 (2.36–2.55)	0.839 (0.756–0.931)
Agricultural town	33,880	2.38 (2.32–2.44)	0.892 (0.824–0.966)
Remote township	31,017	2.35 (2.29–2.42)	0.748 (0.693–0.808)
**Comorbidities (reference: without)**			
Diabetes	156,293	2.40 (2.37–2.43)	**1.138 (1.096–1.181)**
Anxiety	398,918	2.43 (2.41–2.44)	**3.685 (3.587–3.785)**
Cushing’s disease	1169	2.57 (2.23–3.01)	1.374 (0.960–1.966)
Parkinson’s disease	21,716	2.45 (2.38–2.54)	**1.648 (1.509–1.799)**
Alzheimer’s disease	9631	2.31 (2.20–2.42)	**1.685 (1.473–1.927)**
Coronary heart disease	132,622	2.50 (2.47–2.54)	**1.205 (1.159–1.254)**
Breast cancer	10,417	2.46 (2.35–2.57)	**1.280 (1.131–1.449)**
Human immunodeficiency virus	2458	2.36 (2.16–2.60)	**2.283 (1.763–2.955)**

Adjustment for gender, age, low–income, urbanization level, comorbidities. IRR, incidence rate ratio; CI, confidence interval. Bold text indicates statistical significance. (*p* < 0.05).

**Table 4:** Subgroups Poisson regression analysis of Ultraviolet radiation b (UVB) and depression.

	UVB WHO Level (Reference: Low Level)			
	Adjusted IRR (95%CI)			
	Moderate Level	High Level	Very High Level	Extreme Level
**Gender**				
**Female**	**0.885 (0.816–0.959)**	1.030 (0.896–1.184)	**1.697 (1.439–2.000)**	**2.825 (2.381–3.352)**
**Male**	**0.899 (0.814–0.993)**	**1.305 (1.113–1.531)**	**1.731 (1.411–2.123)**	**2.749 (2.227–3.393)**
**Low income**				
Yes	**0.888 (0.817–0.966)**	1.120 (0.968–1.297)	**1.823 (1.529–2.174)**	**2.780 (2.300–3.359)**
No	**0.884 (0.805–0.972)**	1.153 (0.992–1.340)	**1.593 (1.321–1.921)**	**2.808 (2.331–3.383)**
**Urbanization level**				
Highly urbanized				
Yes	–	–	–	–
**No**	**0.895 (0.844–0.949)**	**1.151 (1.040–1.275)**	**1.742 (1.536–1.976)**	**2.828 (2.482–3.223)**
**Comorbidities**				
Diabetes				
Yes	0.881 (0.765–1.015)	1.17 (0.917–1.494)	**1.639 (1.204–2.231)**	**2.683 (1.971–3.652)**
**No**	**0.887 (0.827–0.951)**	**1.122 (0.999–1.26)**	**1.730 (1.503–1.993)**	**2.828 (2.441–3.276)**
Anxiety				
**Yes**	**0.804 (0.730–0.885)**	1.159 (0.999–1.344)	**1.699 (1.407–2.052)**	**2.821 (2.323–3.426)**
**No**	**0.956 (0.881–1.038)**	**1.118 (0.964–1.296)**	**1.734 (1.457–2.065)**	**2.806 (2.339–3.365)**
Cushing’s disease				
Yes	0.792 (0.122–5.16)	–	–	2.245 (0.094–53.642)
**No**	**0.889 (0.835–0.947)**	**1.138 (1.025–1.264)**	**1.717 (1.511–1.952)**	**2.781 (2.435–3.177)**
Parkinson’s disease				
Yes	0.728 (0.501–1.06)	1.017 (0.585–1.77)	1.062 (0.38–2.97)	2.157 (1.179–3.945)
**No**	**0.896 (0.84–0.954)**	**1.139 (1.024–1.268)**	**1.727 (1.518–1.965)**	**2.844 (2.482–3.259)**
Alzheimer’s disease				
Yes	0.623 (0.357–1.088)	0.803 (0.343–1.879)	1.274 (0.436–3.725)	–
**No**	**0.895 (0.84–0.953)**	**1.142 (1.027–1.269)**	**1.714 (1.506–1.95)**	**2.788 (2.441–3.184)**
Coronary heart disease				
Yes	**0.824 (0.707–0.960)**	1.078 (0.830–1.400)	**1.727 (1.294–2.304)**	**2.108 (1.479–3.004)**
**No**	**0.901 (0.841–0.965)**	**1.137 (1.015–1.275)**	**1.701 (1.474–1.962)**	**2.929 (2.538–3.381)**
Breast cancer				
Yes	0.933 (0.43–2.023)	–	1.058 (0.243–4.598)	**4.894 (1.414–16.936)**
**No**	**0.889 (0.835–0.947)**	**1.140 (1.026–1.266)**	**1.717 (1.509–1.952)**	**2.770 (2.424–3.165)**
Human immunodeficiency virus				
Yes	0.536 (0.071–4.070)	–	**9.134 (1.196–69.763)**	5.557 (0.161–191.663)
**No**	**0.889 (0.835–0.947)**	**1.134 (1.021–1.259)**	**1.709 (1.503–1.943)**	**2.777 (2.431–3.172)**

Adjustment for gender, age, low–income, urbanization level, comorbidities. IRR, incidence rate ratio; CI, confidence interval. Bold text indicates statistical significance. (*p* < 0.05).

## Data Availability

Research has limited the availability of these data. The study data were obtained from NHRID with permission from the National Health Insurance Administration of Taiwan, where permission was obtained from the authors.
